# Tannic Acid Mitigates Monosodium Glutamate‐Induced Cortical Damage by Modulating Synaptic and Calcium‐Related Gene and Protein Expression

**DOI:** 10.1002/jat.70185

**Published:** 2026-03-27

**Authors:** Medine Sibel Karagac, Enver Fehim Koçpinar, Hamid Ceylan

**Affiliations:** ^1^ Department of Molecular Biology and Genetics, Faculty of Science Atatürk University Erzurum Türkiye; ^2^ Department of Medical Laboratory Techniques, Vocational School of Health Services Muş Alparslan University Muş Türkiye; ^3^ East Anatolian High Technology Research and Application Center (DAYTAM), Atatürk University Erzurum Türkiye

**Keywords:** cerebral cortex, excitotoxicity, monosodium glutamate, synaptic proteins, tannic acid

## Abstract

Neurotoxicity induced by excessive glutamatergic signaling is associated with synaptic dysfunction, calcium imbalance, and oxidative stress, which are key molecular events implicated in several neurodegenerative conditions. Monosodium glutamate (MSG), a common flavor enhancer, may exert neurotoxic effects, particularly on synaptic integrity, though mechanisms remain unclear. Tannic acid (TA), a natural polyphenol, has been proposed as a neuroprotective compound. This study investigated the impact of MSG on synaptic components beyond classical AD markers and assessed the protective potential of TA. Rats were randomly divided into four groups (*n* = 6 per group) and treated with MSG (2 g/kg) and/or TA (50 mg/kg) by oral gavage for 21 consecutive days. Gene and protein expression levels of the synaptic markers (GRIN2A, GRIN2B, DLG2, SNAP25, SCN2A, and ATP2B2) in the cerebral cortex were analyzed using qPCR and western blot. MSG treatment significantly downregulated SNAP25, GRIN2B, DLG2, and SCN2A at both mRNA and protein levels, indicating synaptic dysfunction. GRIN2A and ATP2B2 showed reduced mRNA expression, but protein levels were inconsistent. MSG+TA group showed no significant difference compared with the control group, while TA alone produced minimal changes, suggesting that its role is primarily protective under toxic stress. These findings suggest that chronic MSG exposure disrupts synaptic molecular architecture, whereas the restorative effect of TA may be attributed to its ability to modulate MSG‐induced molecular alterations. The data emphasize synaptic pathways as alternative neurotoxicity targets and highlight TA's potential in mitigating diet‐related excitotoxic synaptic alterations. Further functional and pathway‐based studies are needed to confirm the underlying mechanisms.

## Abstract 2

Chronic monosodium glutamate (MSG) exposure disrupted key synaptic proteins (SNAP25, GRIN2B, DLG2, and SCN2A) in the rat cerebral cortex, indicating excitotoxic synaptic impairment beyond classical neurodegeneration markers. Tannic acid (TA) largely prevented these MSG‐induced molecular alterations while showing minimal effects alone. These findings highlight synaptic architecture as a primary target of MSG‐related neurotoxicity and suggest a stress‐dependent neuroprotective role for TA. Further functional studies are required to clarify the underlying mechanisms.

## 1 Introduction

Recent studies have highlighted the critical role of dietary habits in the onset and progression of complex neurodegenerative conditions (Ellouze et al. 2023). Among these, poor adherence to antioxidant‐rich diets and excessive consumption of saturated fats, refined sugars, or processed foods has been associated with increased cognitive decline and accelerated neurodegenerative changes (Morris et al. 2015; Solfrizzi et al. 2017). Certain dietary components may directly influence neuronal health by triggering mechanisms such as oxidative stress, neuroinflammation, and synaptic dysfunction (Butterfield and Halliwell 2019). Additionally, nutritional deficiencies—particularly in vitamins B12, D, E, and omega‐3 fatty acids—have been shown to impair synaptic plasticity and increase amyloid‐β accumulation in vulnerable brain regions (Cabal‐Herrera et al. 2025; Mou et al. 2023). As a result, dietary modulation is increasingly recognized as a promising avenue for both prevention and therapeutic intervention in neurodegeneration.

Glutamate is the principal excitatory neurotransmitter in the central nervous system and plays a critical role in synaptic transmission, neuronal plasticity, learning, and memory (Tamagno and Freeman 2025; Wang et al. 2024). However, excessive glutamatergic stimulation leads to excitotoxicity, a pathological process characterized by calcium overload, oxidative stress, mitochondrial dysfunction, and ultimately neuronal damage. These events disrupt synaptic integrity and are considered key molecular features of neurotoxicity (Misrani et al. 2021). Monosodium glutamate (MSG), a widely used food additive, has been extensively employed in experimental models to induce glutamate‐mediated excitotoxicity (Altintas et al. 2025; Hajihasani et al. 2020; Baytar et al. 2026). High doses of MSG used in experimental studies are considerably higher than typical human dietary exposure and are intended to reproduce excitotoxic conditions within a limited time frame (Niaz et al. 2018). The Joint FAO/WHO Expert Committee on Food Additives (JECFA) has classified MSG as a food additive with an acceptable daily intake (ADI) “not specified,” indicating a high margin of safety at normal consumption levels, while the European Food Safety Authority (EFSA) has proposed a group ADI of 30 mg/kg body weight for glutamates (Walker and Lupien 2000; Zanfirescu et al. 2019). Under physiological conditions, circulating glutamate does not readily cross the blood–brain barrier (BBB) due to the presence of high‐affinity transport systems that tightly regulate its concentration in the central nervous system. However, excessive systemic exposure, metabolic stress, or oxidative damage may indirectly influence brain glutamate homeostasis and promote excitotoxic signaling (Gruenbaum, Schonwald, et al. 2024; Gruenbaum, Zlotnik, et al. 2024; Kim et al. 2024). Therefore, MSG administration at high experimental doses is widely used as a model of glutamate‐mediated neurotoxicity rather than a direct representation of human dietary intake. High‐dose MSG exposure has been reported to impair neuronal homeostasis through increased reactive oxygen species production, altered calcium signaling, and synaptic protein dysregulation, particularly in cortical regions that are highly vulnerable to metabolic and excitatory stress (Asejeje et al. 2024; Kesherwani et al. 2024; Taha et al. 2025). It is known to induce excitotoxicity by overstimulating glutamate receptors, thereby disrupting synaptic integrity (Nicosia et al. 2024). These alterations resemble early synaptic vulnerability patterns reported in neurodegenerative conditions; however, MSG administration represents an excitotoxic stress model rather than a disease‐specific model (Singh and Panda 2024). Therefore, the MSG model provides a useful experimental framework for investigating the molecular basis of excitotoxic neuronal injury and for evaluating potential neuroprotective compounds.

Bioactive compounds from natural sources have increasingly been recognized for their protective effects against neurotoxicity (Li et al. 2017; Swamy et al. 2013). Among them, tannic acid (TA) stands out due to its phenolic structure and functional attributes, including antioxidant activity, metal chelation, and regulation of intracellular signaling pathways (Azimullah et al. 2023; Karadas et al. 2024; Kizir et al. 2023; Ozturk et al. 2024). By mitigating oxidative stress, maintaining neuronal stability, and preserving synaptic communication, TA exhibits strong potential as a neuroprotective agent (Kim et al. 2022). Through these properties, TA has emerged as a promising candidate for attenuating excitotoxic damage and restoring neuronal molecular homeostasis (Karagac and Ceylan 2023).

Although amyloid‐β plaques and neurofibrillary tangles are widely studied in Alzheimer's disease research, increasing evidence suggests that synaptic dysfunction and calcium imbalance are broader mechanisms observed across multiple neurodegenerative contexts (De Strooper and Karran 2016; Musiek and Holtzman 2015). The complexity of ad pathogenesis necessitates a broader exploration of alternative molecular and cellular mechanisms that contributes to disease progression. At this stage, advanced bioinformatics‐based approaches help us to interpret omics data to evaluate the global landscape in gene expression. Identification of aberrantly expressed hub genes can help us understand how complex diseases such as AD are regulated and will provide a powerful framework for determining accurate therapeutic targets and prevention measures (Ceylan 2021a, 2021b).

In our previous study (Ceylan 2022), we employed systems‐level bioinformatics tools to identify a panel of genes intricately involved in synaptic function and neuronal communication. The genes and proteins analyzed in this study were selected based on previous experimental evidence demonstrating their involvement in synaptic transmission, vesicle release, NMDA receptor signaling, and calcium homeostasis under excitotoxic conditions. Rather than applying a systems‐level omics approach, we focused on targeted molecular markers that are known to be sensitive to glutamate‐induced neuronal stress and are functionally associated with cortical synaptic integrity. Among the highlighted targets were *GRIN2A, GRIN2B*, *DLG2*, *SNAP25*, *SCN2A*, and *ATP2B2* genes that regulate synaptic vesicle release, glutamatergic signaling, and neuronal excitability (Tantillo et al. 2023; Zhang et al. 2025). These findings support the view that disruptions in synaptic machinery may represent early and targetable events in neurodegeneration.

The genes and proteins analyzed were selected to represent interconnected elements of synaptic vesicle release, NMDA receptor–mediated signaling, and calcium homeostasis that collectively determine neuronal vulnerability to excitotoxicity, thereby providing an integrated molecular assessment of cortical synaptic integrity under glutamate‐induced stress. The cerebral cortex was chosen due to its intense glutamatergic activity and well‐known susceptibility to metabolic and excitotoxic damage in MSG models, and a 21‐day exposure paradigm was used to reveal sustained rather than acute molecular alterations. Since the combined transcriptional and translational evaluation of synaptic vesicle‐associated proteins and NMDA receptor subunits in this region, together with the modulatory potential of TA, remains limited, this study aimed to fill this toxicological gap. We therefore hypothesized that subchronic MSG exposure would disrupt cortical synaptic and NMDA receptor–related molecular markers, and TA acid treatment would attenuate these changes.

Based on this background, the present study aimed to investigate the effects of TA on MSG‐induced molecular alterations in the rat cerebral cortex by evaluating the expression of key genes and proteins involved in synaptic function and calcium signaling. By focusing on glutamate‐mediated excitotoxic mechanisms, this study seeks to provide molecular evidence for the potential neuroprotective role of TA.

## 2 Materials and Methods

### 2.1 Experimental Design

A total of 24 male Sprague–Dawley rats ( *Rattus norvegicus* , 180 g ± 10 g) were used in this study. Only male rats were used to avoid the possible effects of hormonal fluctuations associated with the estrous cycle on biochemical and molecular parameters and to reduce interindividual variability (Baytar et al. 2024; Baytar et al. 2025). Animals were acclimatized before the experimental procedures and monitored daily for general health status, food and water intake, grooming behavior, and body weight changes. The animals were randomly divided into four groups: Control (Con), MSG, TA, and MSG + TA. The animals were randomly allocated to the experimental groups using a simple randomization method. Due to the nature of the experimental design, blinding during treatment administration was not feasible; however, all samples were coded before molecular analyses to minimize bias. A priori power analysis was not performed to determine the sample size. The number of animals (*n* = 6 per group) was selected based on previous similar studies and commonly used experimental designs in the literature in accordance with ethical considerations. The control group received only saline (0.9%) via oral gavage. The dose of MSG (2 g/kg) was selected based on previous experimental studies demonstrating its neurotoxic potential while remaining within the safety limits reported by the European Food Safety Authority (EFSA) and the No Observed Adverse Effect Level (NOAEL) (Beyreuther et al. 2007; Biney et al. 2022). This dose is higher than the acceptable daily intake defined for humans but is commonly used in experimental models to induce neurotoxicity within a limited period. The TA dose (50 mg/kg) was determined according to earlier studies showing significant antioxidant and neuroprotective effects without inducing systemic toxicity (de Veras et al. 2021; Tuzmen et al. 2015). MSG and TA were administered once daily by oral gavage for 21 consecutive days. To enhance the prophylactic effect of TA, animals in the combined MSG + TA group were pretreated with TA 1 h before MSG administration (Al‐Jaouni et al. 2019). MSG (Merck Millipore, Germany) and TA (Sigma‐Aldrich, Canada) were dissolved in a 0.9% saline solution, and therefore, pH adjustment was not required. All rats were housed in standard plastic cages under controlled environmental conditions (temperature: 22°C ± 3°C; humidity: 55%; 12‐h light/dark cycle), with ad libitum access to standard chow and tap water. On Day 21, immediately after the final treatment, the rats were euthanized under deep general anesthesia induced by intramuscular ketamine (50 mg/kg) and xylazine (10 mg/kg) (Sigma Aldrich, Canada) to ensure adequate sedation, analgesia, and muscle relaxation. Following euthanasia, cerebral cortex tissues were rapidly dissected, rinsed with cold phosphate‐buffered saline (PBS, Thermo Scientific, USA), and stored at −80°C for qPCR and Western blot analyses to assess synaptic markers. The Atatürk University Animal Experiments Local Ethics Committee approved this experimental protocol (protocol no.: 2021–3/63). All experimental procedures were carried out in accordance with the guidelines of the NIH (National Research Council Committee 2011) Guide for the Care and Use of Laboratory Animals. All efforts were made to minimize animal suffering and to reduce the number of animals used. No unexpected mortality or severe clinical signs were observed during the experimental period; therefore, no animal reached predefined humane endpoint criteria. Additionally, to improve transparency, the study design and experiments were reported in accordance with ARRIVE 2.0 guidelines (Percie du Sert et al. 2020) and the 3R/6R principles.

### 2.2 RNA Isolation and cDNA Synthesis

Total RNA was isolated from rat cerebral cortex tissue using the Invitrogen RNA Isolation Kit (Thermo Fisher Scientific, USA) according to the manufacturer's instructions. The purity and concentration of the isolated RNA samples were assessed using a Multiskan GO microplate spectrophotometer (Thermo Fisher Scientific, USA), and RNA integrity was confirmed by A260/A280 ratios (Kansu et al. 2024). First‐strand cDNA synthesis was performed using the iScript cDNA Synthesis Kit (Bio‐Rad Laboratories, USA) according to the supplier's protocol. Equal amounts of total RNA were used for all samples to ensure consistency in downstream analyses.

### 2.3 Quantitative Real‐Time PCR (qPCR)

Quantitative real‐time PCR was carried out using the SsoAdvanced Universal SYBR Green Supermix (Bio‐Rad Laboratories, USA) in a thermal cycler system. Each 20‐μL reaction mixture contained 10 μL of SYBR Green Supermix, 1 μL of each forward and reverse primer, 2 μL of cDNA template, and nuclease‐free water. The thermal cycling conditions were as follows: initial polymerase activation and DNA denaturation at 95°C for 2 min, followed by 40 cycles of denaturation at 95°C for 15 s, annealing, and extension at 60°C for 20 s. All reactions were performed in triplicate. *Gapdh* (NM_017008.3) was used as the reference (housekeeping) gene. Relative gene expression was calculated using the 2^−ΔΔCt method, assuming comparable amplification efficiencies between target and reference genes (Livak and Schmittgen 2001). Primers for target genes were designed using the Primer3 online software (https://primer3.ut.ee/) (accessed on June 15, 2025) (Tosun et al. 2024). Primer specificity was verified in silico using NCBI Primer‐BLAST (https://blast.ncbi.nlm.nih.gov/Blast.cgi, accessed on June 15, 2025) to ensure target‐specific amplification. All primers are listed in Table 1.

**TABLE 1:** Primer sets used in qPCR.

Gene symbol	Accession ID	Sequence	Tm (°C)
*Grin2a_F*	NM_012573.3	^5′‐^CCAGTTTGTTGGTGATGGTG^‐3′^	59.85
*Grin2a_R*		^5′‐^TCTTCTCGTTGTGGCAGATG^‐3′^	59.98
*Grin2b_F*	NM_012574.1	^5′‐^AATGGCGGATAAGGATGAGTC^‐3′^	60.30
*Grin2b_R*		^5′‐^AGGTGGTGACGATGGAAAAG^‐3′^	59.97
*Dlg2_F*	NM_022282.2	^5′‐^ACGAGAGCAGATGATGAACC^‐3′^	59.94
*Dlg2_R*			
		^5′‐^TGACATACAGGGAGCGTTTCT^‐3′^	59.75
*Snap25_F*	NM_001270575.1	^5′‐^ACACCCAGAATCGCCAGAT^‐3′^	60.49
*Snap25_R*		^5′‐^GAGGAGAGCACAGCAGAAGG^‐3′^	60.28
*Scn2a_F*	NM_012647.2	^5′‐^TATCTCCAACCACACGACCA^‐3′^	59.96
*Scn2a_R*			
		^5′‐^TCTCCACACTGCTGCCTATG^‐3′^	60.01
*Atp2b2_F*	NM_012508.5	^5′‐^GGCAGAAGATGAAGGTGAAG^‐3′^	60.20
*Atp2b2_R*			
		^5′‐^ACGAGGACCACACAGATGAC^‐3′^	59.67
*Gapdh_F*	NM_017008.3	^5′‐^AAACCCATCACCATCTTCCA^‐3′^	60.17
*Gapdh_R*			
		^5′‐^ATACTCAGCACCAGCATCACC^‐3′^	60.16

*Note:* F; forward, R; reverse, Tm; melting temperature.

### 2.4 Western Blot Analysis

Protein expression levels of GRIN2A, GRIN2B, DLG2, SNAP25, SCN2A, and ATP2B2 in rat cerebral cortex tissue were analyzed using Western blotting. Brain cortex tissues were homogenized in ice‐cold RIPA lysis buffer (Thermo Fisher Scientific, USA) supplemented with a protease inhibitor cocktail (Sigma‐Aldrich, USA). Total protein concentrations were determined using the Bradford method (Thermo Fisher Scientific, USA) (Bradford 1976). Equal amounts of protein were loaded onto 10%–12% SDS‐polyacrylamide gels and separated by electrophoresis. Proteins were then transferred onto PVDF membranes (Millipore, USA) using a semidry blotting system. Membranes were blocked with 5% nonfat dry milk in TBS‐T buffer (20 mM of Tris‐HCl, 150 mM NaCl, 0.1% Tween‐20, pH 7.4, Sigma Aldrich, Canada) for 1 h at room temperature to prevent nonspecific binding. Membranes were incubated overnight at 4°C with the primary antibodies (diluted in TBS‐T with 1% BSA (Sigma Aldrich) or milk as appropriate). Protein bands were visualized using an enhanced chemiluminescence (ECL) detection system (Bio‐Rad Laboratories, USA), and images were captured using a ChemiDoc MP Imaging System (Bio‐Rad Laboratories, USA). Densitometric analysis of the bands was performed using ImageJ software (NIH, USA). Protein bands were identified according to their expected molecular weights using a prestained protein ladder (GeneDirex, #PM007‐0500) and manufacturer‐provided antibody datasheets. Each membrane was probed with a single primary antibody; therefore, stripping was not performed. Western blot analyses were performed using samples obtained from independent animals (biological replicates). Protein expression levels of target genes were normalized to β‐Actin (1:10,000; E‐AB‐40338, Elabscience, USA) and expressed as relative values compared to the control group. In the present study, GRIN2A (1:1000; #E‐AB‐40338, Elabscience, USA), GRIN2B (1:1000; #E‐AB‐40338, Elabscience, USA), SNAP25 (1:1000; #E‐AB‐40338, Elabscience, USA), ATP2B2 (1:1000; #PA1‐915, ThermoFisher Scientific, USA), SCN2A (1:1000; #PA5‐36545, ThermoFisher Scientific, USA), and DLG2 (1:1000; #bs‐3348R, Bioss, USA) primary antibodies were used. Goat antirabbit IgG (1:1000; #bs‐0295G‐HRP, Bioss, USA) was used as a secondary antibody.

### 2.5 Statistical Analysis

A one‐way ANOVA followed by Dunnett's post hoc test was used for comparisons versus the control group. In addition, a predefined comparison between the MSG and MSG+TA groups was performed to directly test the protective effect of TA against MSG‐induced changes. MGS and MSG+TA groups were compared using a parametric unpaired *t*‐test. Statistical comparisons were made using GraphPad Prism (Software, San Diego, CA) software. Significant differences between groups (compared to the control group) are indicated with asterisks. The statistically significant differences are presented as follows: ^ns^ *p* > 0.05 (not significant), */•*p* < 0.05 (significant), **/••*p* < 0.01 (very significant), ***/•••*p* < 0.001 or 0.0001 (extremely significant). Dunnet's multiple comparison test significance levels are marked with (•), and *t*‐test significance levels are marked with (*).

## 3 Results

*Grin2a* mRNA levels were significantly downregulated in the cortex of MSG‐treated rats, whereas no significant change was observed in the TA group. In the MSG+TA co‐treatment group, *Grin2a* mRNA levels increased compared to the MSG group and approached control levels (Figure 1A). Interestingly, western blot analysis revealed that GRIN2A protein levels were elevated in the MSG group, contrary to the mRNA expression pattern. Protein levels remained unchanged in the TA‐alone group and were not significantly different from the control group in the MSG+TA co‐treatment group (Figure 1B). The discrepancy between GRIN2A mRNA and protein expression under MSG exposure suggests potential posttranscriptional modifications or translational compensation. TA may contribute to rebalancing MSG‐induced expression alterations.

**FIGURE 1:**
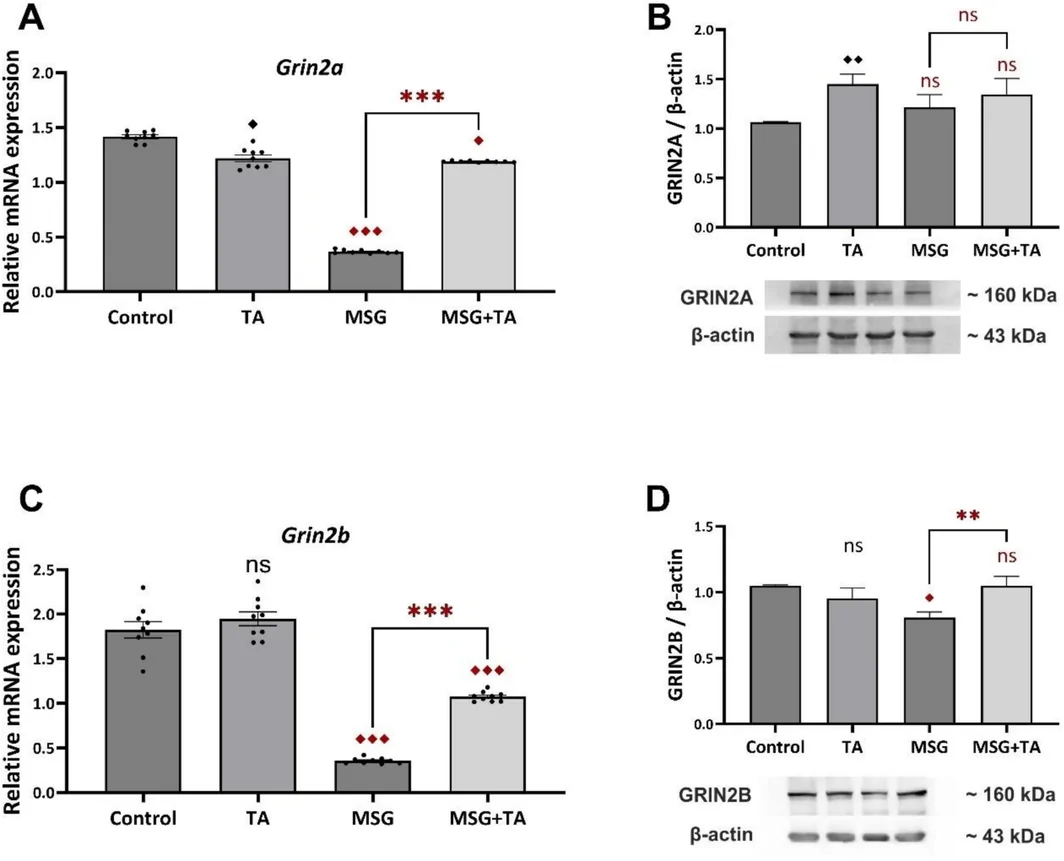
Effects of MSG, TA, and their combination on GRIN2A and GRIN2B expression in the rat cortex. (A) Relative mRNA expression levels of *Grin2a* were assessed by qRT‐PCR in control, MSG‐, TA‐, and MSG+TA‐treated groups. (B) Quantification of GRIN2A protein levels and representative western blot image in cortical tissue. (C) Quantitative RT‐PCR analysis of *Grin2b* mRNA expression in cortical tissue of control, MSG‐, TA‐, and MSG+TA‐treated rats. (D) Representative western blot image and corresponding quantification of GRIN2B protein levels. Diamond symbols (♦) indicate comparisons versus the control group (Dunnett's test), while asterisks (*) represent planned comparisons between the MSG and MSG+TA groups (*t*‐test). Symbols denote significance levels as follows: ^ns^ *p* > 0.05 (not significant) ♦ or * *p* < 0.05 (significant), ♦♦ or ** *p* < 0.01 (very significant), ♦♦♦ or *** *p* < 0.001 (extremely significant), according to GraphPad Prism output. Data are shown as the mean ± SEM.

*Grin2b* mRNA levels were significantly downregulated in the MSG‐treated group, whereas the TA‐treated group showed no significant changes. TA significantly increased GRIN2B expression compared with the MSG group, and the values were not significantly different from the control group (Figure 1C). Protein analysis via western blotting also revealed a parallel trend, with decreased GRIN2B levels in the MSG group and normalization in the MSG+TA group (Figure 1D). GRIN2B downregulation implies glutamatergic signaling impairment induced by MSG, which appears to be counteracted by TA, indicating a potential protective mechanism at the receptor level.

*Dlg2* mRNA expression was significantly downregulated in the MSG‐treated group but remained unchanged in the TA‐only group. The combined MSG+TA treatment restored *Dlg2* expression to levels comparable to the control group (Figure 2A). Protein expression trends were consistent with mRNA data. The suppression of DLG2 by MSG may reflect impaired synaptic scaffolding, which is alleviated by the co‐administration of TA, suggesting a modulatory effect on synaptic structural protein expression (Figure 2B).

**FIGURE 2:**
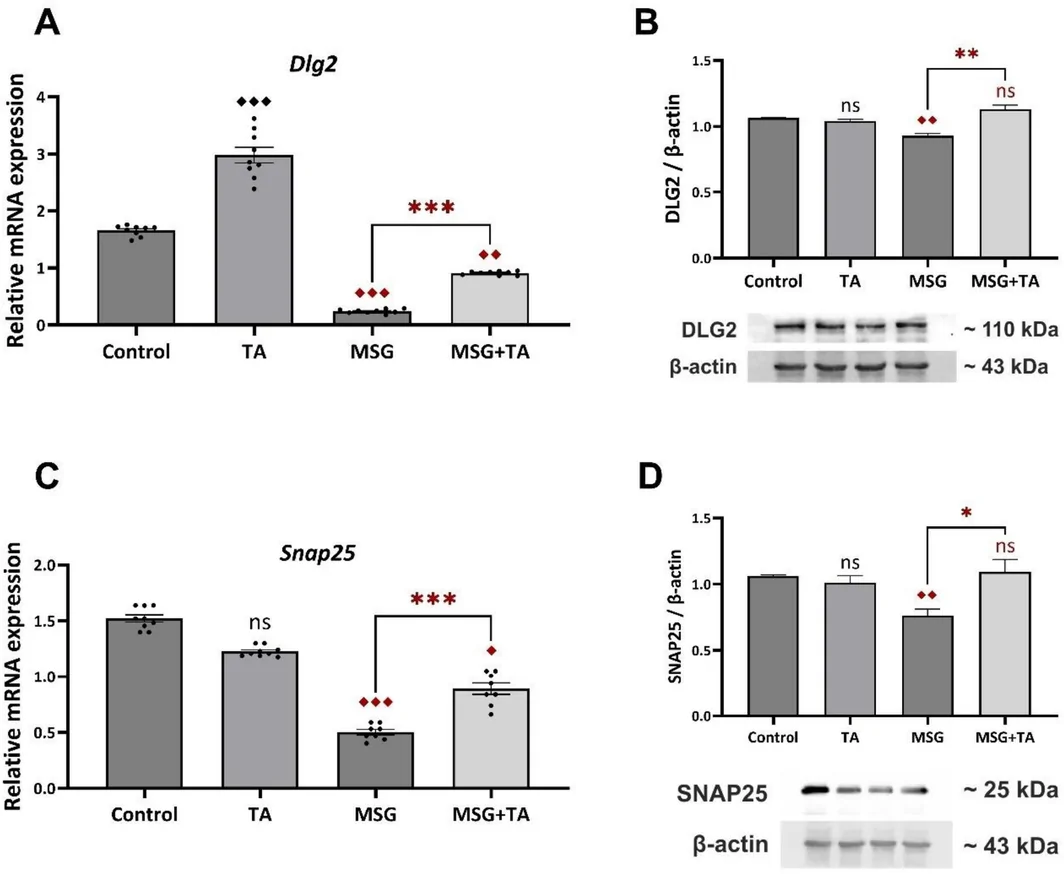
Effects of MSG, TA, and their combination on DLG2 and SNAP25 expression in the rat cortex. (A) Relative mRNA expression levels of *Dlg2* were assessed by qRT‐PCR in control, MSG‐, TA‐, and MSG+TA‐treated groups. (B) Quantification of DLG2 protein levels and representative western blot image in cortical tissue. (C) Quantitative RT‐PCR analysis of *Snap25* mRNA expression in cortical tissue of control, MSG‐, TA‐, and MSG+TA‐treated rats. (D) Representative western blot image and corresponding quantification of SNAP25 protein levels. Diamond symbols (♦) indicate comparisons versus the control group (Dunnett's test), while asterisks (*) represent planned comparisons between the MSG and MSG+TA groups (*t*‐test). Symbols denote significance levels as follows: ^ns^ *p* > 0.05 (not significant) ♦ or * *p* < 0.05 (significant), ♦♦ or ** *p* < 0.01 (very significant), ♦♦♦ or *** *p* < 0.001 (extremely significant), according to GraphPad Prism output. Data are shown as the mean ± SEM.

A significant decrease in *Snap25* mRNA expression was observed in the cortex of rats treated with MSG alone, while no significant change was detected in the TA‐treated group. Co‐treatment with MSG and TA significantly increased *Snap25* expression compared with the MSG group, and the values were not significantly different from the control group (Figure 2C). Western blot analysis showed that the protein levels mirrored the mRNA expression, with reduced levels in the MSG group, unchanged levels in the TA group, and near‐control levels in the MSG+TA group (Figure 2D). The reduction in SNAP25 expression following MSG exposure suggests synaptic dysfunction, which may be ameliorated by the neuroprotective properties of TA.

A marked reduction in *Scn2a* mRNA expression was detected in the MSG group, while TA alone had no effect. MSG+TA co‐treatment significantly increased *Scn2a* mRNA and protein levels compared to MSG alone, restoring them close to baseline (Figure 3A). Protein data were consistent with mRNA trends (Figure 3B). SCN2A downregulation indicates disrupted sodium channel function due to MSG, which may impair neuronal excitability; this effect is seemingly mitigated by TA, suggesting neuroprotection.

**FIGURE 3:**
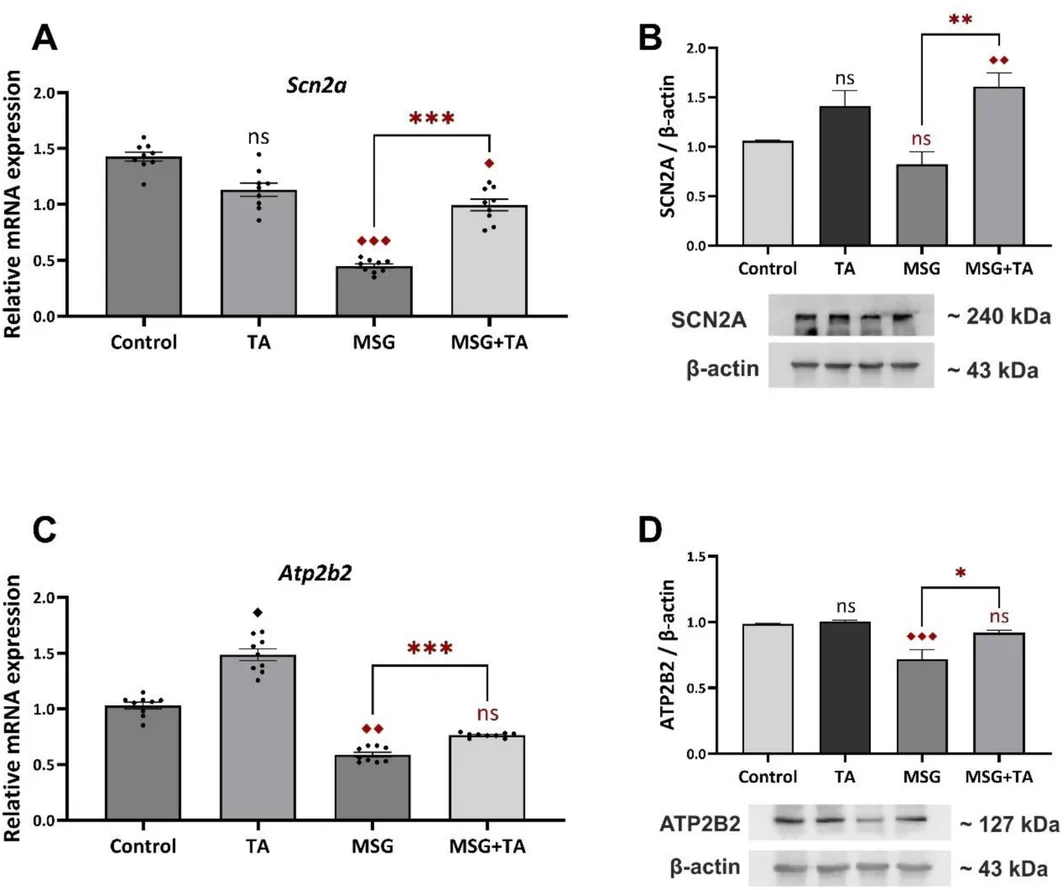
Effects of MSG, TA, and their combination on SCN2A and ATP2B2 expression in the rat cortex. (A) Relative mRNA expression levels of *Scn2a* were assessed by qRT‐PCR in control, MSG‐, TA‐, and MSG+TA‐treated groups. (B) Quantification of SCN2A protein levels and representative western blot image in cortical tissue. (C) Quantitative RT‐PCR analysis of *Atp2b2* mRNA expression in cortical tissue of control, MSG‐, TA‐, and MSG+TA‐treated rats. (D) Representative western blot image and corresponding quantification of ATP2B2 protein levels. Diamond symbols (♦) indicate comparisons versus the control group (Dunnett's test), while asterisks (*) represent planned comparisons between the MSG and MSG+TA groups (*t*‐test). Symbols denote significance levels as follows: ^ns^ *p* > 0.05 (not significant) ♦ or * *p* < 0.05 (significant), ♦♦ or ** *p* < 0.01 (very significant), ♦♦♦ or *** *p* < 0.001 (extremely significant), according to GraphPad Prism output. Data are shown as the mean ± SEM.

*Atp2b2* mRNA levels were significantly downregulated in rats treated with MSG alone, with no significant changes observed in the TA‐only group. Co‐treatment brought mRNA levels closer to the control (Figure 3C). In contrast, protein levels were elevated in the MSG group, suggesting a divergence from mRNA expression patterns, and normalized in the MSG+TA group (Figure 3D). The elevated expression of ATP2B2 under MSG stress may reflect a compensatory mechanism for disturbed calcium homeostasis, which appears to be stabilized by TA treatment.

## 4 Discussion

The present study investigated the effects of TA on MSG‐induced alterations in synaptic and calcium‐related molecular markers in the rat cortex. Rather than assessing functional or behavioral outcomes, our findings demonstrate that MSG exposure is associated with significant changes in the expression of synaptic vesicle proteins, NMDA receptor subunits, ion channel components, and calcium extrusion systems at both mRNA and protein levels. Co‐administration of TA attenuated several of these expression‐level alterations. These data therefore reflect molecular modulation under excitotoxic conditions rather than functional recovery.

In recent years, the impact of environmental influences on synaptic health and neurodegeneration has garnered substantial attention (Ceylan and Erdogan 2017). Diet‐derived compounds can modulate neuronal health by regulating oxidative stress, neurotransmitter balance, neuroinflammation, and synaptic integrity through alterations in gene expression. Diets high in excitotoxic additives like MSG have been associated with oxidative stress and impaired neurotransmission (Abushikha et al. 2025; Niaz et al. 2018), while polyphenolic compounds such as TA have demonstrated neuroprotective effects by modulating inflammatory responses, reducing oxidative damage, and preserving synaptic structure (Minocha et al. 2022). Against this background, our study investigated how MSG and TA affect the expression of six synaptic genes (GRIN2A, GRIN2B, DLG2, SNAP25, SCN2A, and ATP2B2) that are interrelated (Figure 4) and densely expressed in brain tissue (Figure 5), within the rat cortex, and evaluated their relevance to synaptic vulnerability under excitotoxic conditions.

**FIGURE 4:**
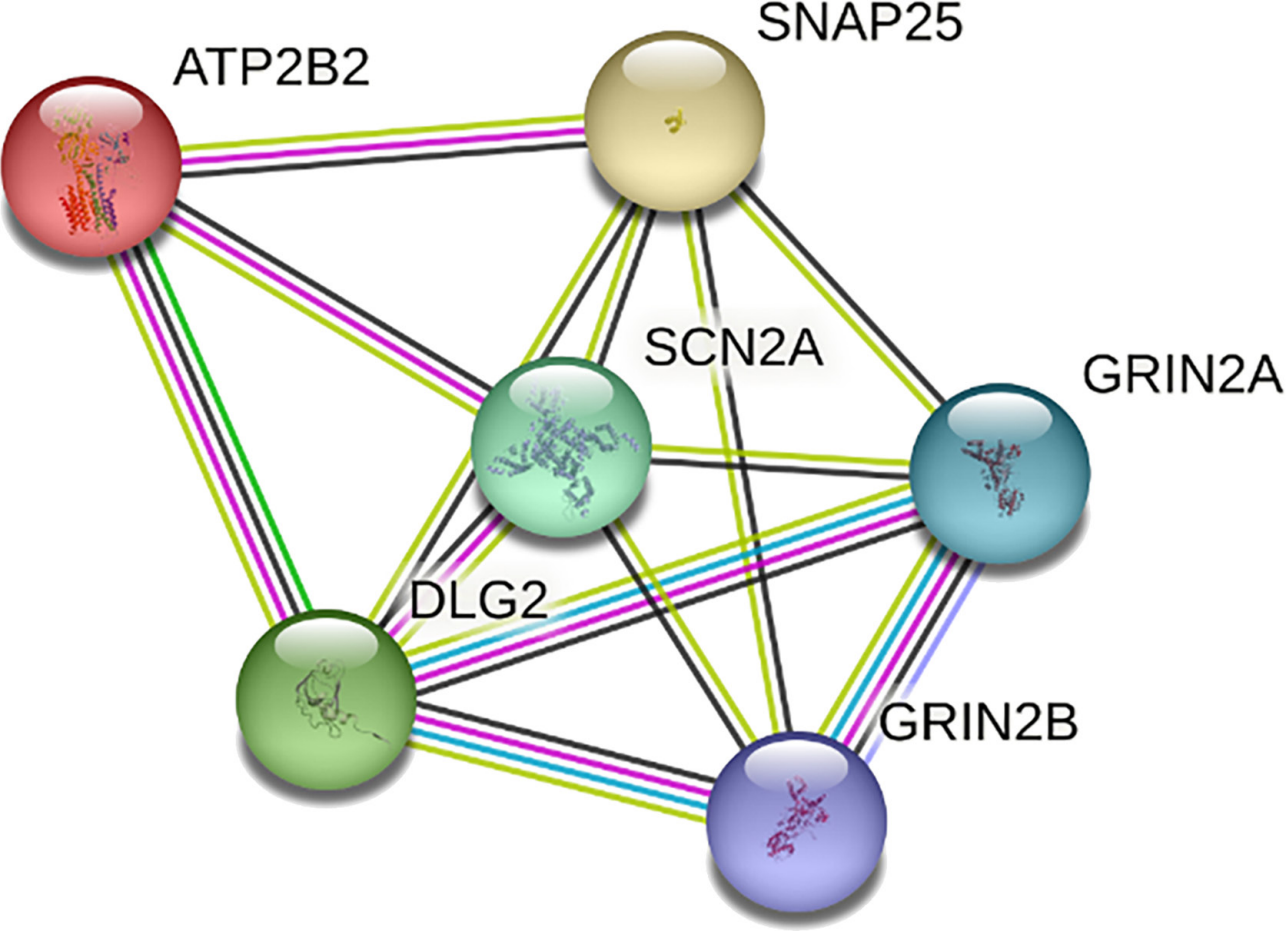
Protein–protein interaction (PPI) network showing the relationships between the genes investigated in the study. The Search Tool for the Retrieval of Interacting Genes/Proteins (STRING) database (https://string‐db.org/) was used to construct the network.

**FIGURE 5:**
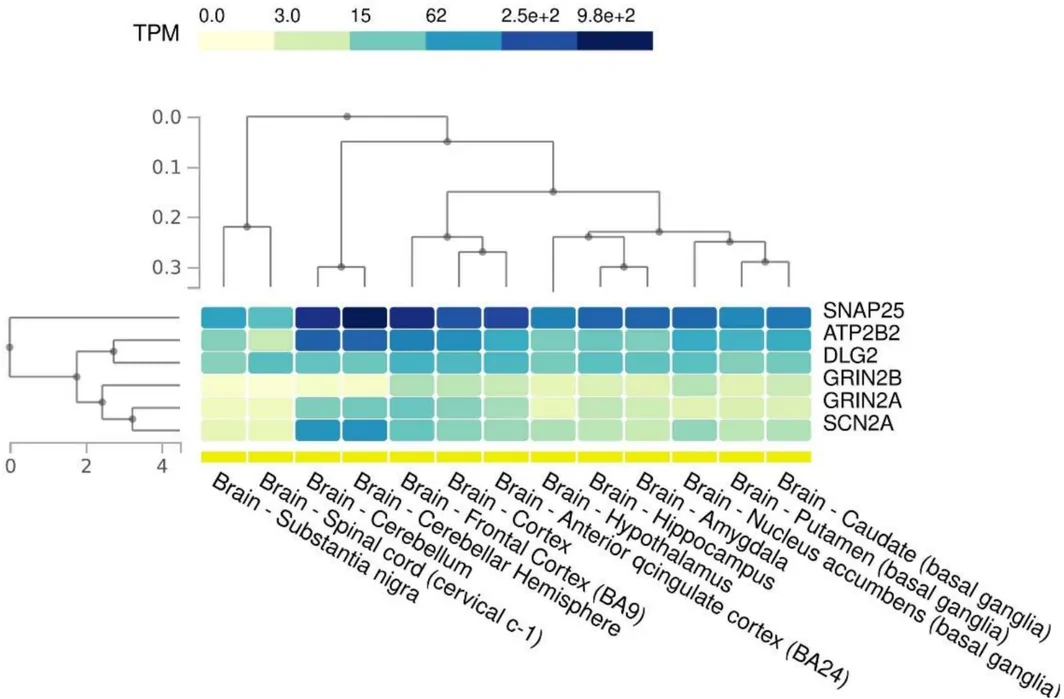
Gene level expressions of SNAP25, GRIN2A, GRIN2B, DLG2, ATP2B2, and SCN2A gene across brain tissue sections available in genotype‐tissue expression (GTEx) database. All data visualization was performed using http://www.gtexportal.org/. TPM; transcripts per million.

Synaptic vesicle dynamics and postsynaptic scaffolding proteins are essential for efficient neurotransmission and neuronal plasticity (Selkoe 2002). Synaptosome‐associated protein 25 (SNAP25) is a critical component of the SNARE complex, facilitating vesicular fusion and neurotransmitter release (Gonzalez‐Lozano et al. 2020), and plays a critical role in synaptic vesicle docking and fusion. Reduced SNAP25 expression is associated with impaired synaptic transmission and synaptic protein loss, reflecting compromised vesicle cycling and neuronal communication (Gustus et al. 2018; Wang et al. 2023). Such downregulation is therefore considered detrimental for synaptic integrity, whereas its restoration supports the maintenance of synaptic function. Another key component, discs large MAGUK scaffold protein 2 (DLG2, also known as PSD‐93), is involved in the structural organization of the postsynaptic density and receptor clustering. Loss of DLG2 disrupts NMDA receptor complex stability and weakens synaptic strength, leading to impaired synaptic plasticity and cognitive performance (Frank et al. 2016). Conversely, maintaining its expression supports postsynaptic integrity (Yu et al. 2017). The MSG‐induced downregulation of these proteins observed in the present study suggests impaired synaptic communication and structural destabilization. The restoration of their expression levels by TA indicates preservation of synaptic function and highlights its modulatory effect on proteins directly involved in neurotransmitter release and postsynaptic signaling.

Glutamate ionotropic receptor NMDA type subunits GRIN2A and GRIN2B, which encode the GluN2A and GluN2B subunits, are central regulators of synaptic plasticity, learning, and memory through calcium‐dependent signaling and balanced NMDA receptor activity (Bading 2017; Drissi et al. 2021; Hu et al. 2016). Alterations in their expression disturb calcium homeostasis promote excitotoxicity and contribute to synaptic dysfunction and cognitive decline (Hansen et al. 2021; Meesawatsom et al. 2020; Vieira et al. 2020). Experimental evidence from disease models indicates that GRIN2A alterations may influence synaptic stability under pathological stress conditions (Du et al. 2021; Raich et al. 2024). Likewise, reduced GRIN2B expression or function has been linked to impaired NMDA receptor signaling and cognitive dysfunction (Li et al. 2009; Yu et al. 2025). In the present study, MSG exposure resulted in a significant decrease in Grin2a mRNA levels despite an increase in GRIN2A protein expression, suggesting the presence of post‐transcriptional regulation or translational compensation under excitotoxic conditions. Such a divergence between mRNA and protein levels may reflect an adaptive response aimed at maintaining synaptic NMDA receptor function. TA co‐treatment restored both mRNA and protein levels toward control values, indicating a stabilizing effect on physiological expression balance. In contrast, both Grin2b mRNA and GRIN2B protein levels were reduced in the MSG group and normalized by TA administration. GRIN2B‐containing NMDA receptors are strongly associated with extrasynaptic signaling and calcium overload–mediated excitotoxicity. Their downregulation under MSG exposure may represent a protective adaptive mechanism to limit excessive calcium influx. The increase in GRIN2B expression in the MSG+TA group compared with the MSG group indicates that TA attenuates the MSG‐induced alteration at the receptor subunit level. Together, these findings indicate differential changes in NMDA receptor subunit expression in response to MSG, which were significantly attenuated by TA treatment.

In addition to vesicle‐associated proteins, ion channel activity and calcium homeostasis are indispensable for neuronal excitability and synaptic transmission. Sodium voltage‐gated channel alpha subunit 2 (SCN2A) encodes a voltage‐gated sodium channel subunit that is essential for action potential initiation and propagation (Baumgartner et al. 2023), whereas ATPase plasma membrane Ca^2+^ transporting 2 (ATP2B2) is responsible for calcium extrusion from neurons and maintenance of intracellular calcium balance. Disruption of these systems by MSG may lead to altered membrane excitability (Ma et al. 2022; Shin et al. 2019; Zhang et al. 2021) and calcium overload (Brendel et al. 2014), which are key features of excitotoxic neuronal damage. Additional studies with knockout (*Scn2a* ^−/−^) and heterozygous haploinsufficient mouse models (*Scn2a* ^+/−^) have demonstrated that *Scn2a* deficiency can lead to massive neuronal apoptosis beyond the loss of synaptic function (Planells‐Cases et al. 2000; Spratt et al. 2019). Several lines of evidence also suggest that calcium‐binding proteins, such as ATP2B2, may become significantly reduced in the aging brain, resulting in altered intracellular calcium balance, which may promote protein aggregation and neuronal vulnerability under pathological conditions (Isaacs et al. 2006). These findings suggest that the normalization of SCN2A and ATP2B2 expression in the TA‐treated group suggests that its neuroprotective action also involves stabilization of ionic homeostasis and prevention of calcium‐mediated toxicity.

Present results are consistent with previously described indirect mechanisms linking systemic MSG exposure to central excitotoxic stress (Dong et al. 2009; Zanfirescu et al. 2019). Thus, the synaptic alterations in our study are likely driven by these secondary excitotoxic mechanisms. TA‐mediated restoration of synaptic protein expression supports its role in preserving synaptic integrity, probably through modulation of oxidative and inflammatory pathways (Salman et al. 2020). In a previous study using the same experimental paradigm (Karagac and Ceylan 2023), we demonstrated modulation of oxidative stress markers (GSH, SOD, and MDA). However, such parameters were not evaluated in the present work, which focuses specifically on synaptic molecular alterations. Together with earlier reports on the neuroprotective effects of polyphenols (Spencer 2010), these data indicate that synaptic system components remain dynamically associated with changes in response to environmental and neurotoxic stimuli (Figure 6).

**FIGURE 6:**
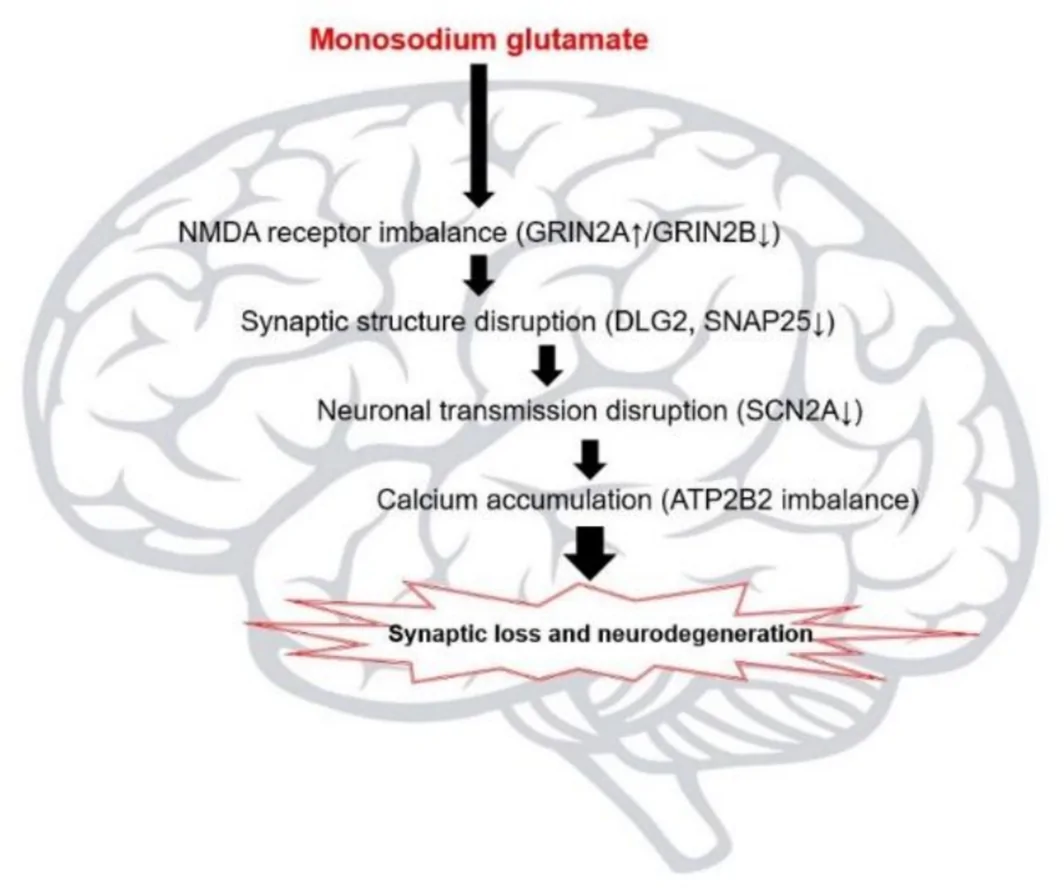
Schematic illustration showing the effects of MSG and TA in the synaptic machinery. Our experiments lead us to the following model: MSG → NMDA receptor imbalance (GRIN2A↑/GRIN2B↓) → Synaptic structure disruption (DLG2, SNAP25↓) → Neuronal transmission disruption (SCN2A↓) → Calcium accumulation (ATP2B2 imbalance).

Our results are in agreement with previous studies reporting the neuroprotective effects of polyphenolic compounds against MSG‐induced neuronal damage. However, unlike earlier studies that mainly focused on biochemical oxidative stress markers, the present study suggests the coordinated regulation of synaptic vesicle proteins, NMDA receptor subunits, ion channel components, and calcium extrusion systems at both mRNA and protein levels. This integrative molecular approach provides a more comprehensive understanding of the mechanisms underlying TA‐mediated neuroprotection.

It is important to emphasize that the present study does not represent a disease‐specific model such as Alzheimer's disease. No amyloid‐β or tau pathology, behavioral assessments, or age‐related disease parameters were evaluated. Instead, the MSG paradigm was employed as an experimental model of glutamate‐mediated excitotoxic stress to investigate synaptic and calcium‐related molecular alterations in the cortex. Therefore, our findings should be interpreted within the framework of excitotoxic neurotoxicity rather than as evidence of disease modeling.

## 5 Conclusion

The combined evaluation of transcriptional and translational changes in multiple synaptic and calcium homeostasis–related targets represents a major strength of the present study, as it allows a more detailed assessment of the functional integrity of neuronal signaling machinery. Importantly, this study does not represent a disease‐specific model such as Alzheimer's disease; rather, it investigates molecular alterations under experimentally induced excitotoxic stress.

Some limitations of this study should be acknowledged. An a priori power analysis was not performed for sample size determination, and only male rats were included to avoid hormonal variability. Furthermore, this study did not include behavioral testing, aged animals, or disease‐specific pathological markers such as amyloid‐β or tau. Future studies including both sexes and functional outcome measures would provide further insight into the neuroprotective potential of TA.

## Author Contributions

**Medine Sibel Karagac:** methodology, investigation. **Enver Fehim Koçpınar:** methodology, formal analysis, data curation. **Hamid Ceylan:** conceptualization, supervision, writing – original draft, writing – review and editing.

## Conflicts of Interest

The authors declare no conflicts of interest.

## Declaration of Generative AI and AI‐Assisted Technologies in the Writing Process

AI‐assisted tools were used solely for language editing and improving readability. These tools were not used for data analysis, interpretation, or generation of scientific content. All scientific decisions and the final version of the manuscript were reviewed and approved by the authors.

## Data Availability

Data will be made available on request.
